# Integration of Antiretroviral Therapy Services into Antenatal Care Increases Treatment Initiation during Pregnancy: A Cohort Study

**DOI:** 10.1371/journal.pone.0063328

**Published:** 2013-05-16

**Authors:** Kathryn Stinson, Karen Jennings, Landon Myer

**Affiliations:** 1 Centre for Infectious Disease Epidemiology and Research, School of Public Health and Family Medicine, University of Cape Town, Cape Town, South Africa; 2 Specialised Health, City Health Directorate, Cape Town, South Africa; Tulane University, United States of America

## Abstract

**Objectives:**

Initiation of antiretroviral therapy (ART) during pregnancy is critical to promote maternal health and prevent mother-to-child HIV transmission (PMTCT). The separation of services for antenatal care (ANC) and ART may hinder antenatal ART initiation. We evaluated ART initiation during pregnancy under different service delivery models in Cape Town, South Africa.

**Methods:**

A retrospective cohort study was conducted using routinely collected clinic data. Three models for ART initiation in pregnancy were evaluated ART ‘integrated’ into ANC, ART located ‘proximal’ to ANC, and ART located some distance away from ANC (‘distal’). Kaplan-Meier methods and Poisson regression were used to examine the association between service delivery model and antenatal ART initiation.

**Results:**

Among 14 617 women seeking antenatal care in the three services, 30% were HIV-infected and 17% were eligible for ART based on CD4 cell count <200 cells/µL. A higher proportion of women started ART antenatally in the integrated model compared to the proximal or distal models (55% vs 38% vs 45%, respectively, global p = 0.003). After adjusting for age and gestation at first ANC visit, women who at the integrated service were significantly more likely to initiate ART antenatally (rate ratio 1.33; 95% confidence interval: 1.09–1.64) compared to women attending the distal model; there was no difference between the proximal and distal models in antenatal ART initiation however (p = 0.704).

**Conclusions:**

Integration of ART initiation into ANC is associated with higher levels of ART initiation in pregnancy. This and other forms of service integration may represent a valuable intervention to enhance PMTCT and maternal health.

## Introduction

Lifelong triple-drug antiretroviral therapy (ART) initiation in eligible HIV-infected women during pregnancy is an important intervention both for preventing mother-to-child transmission (PMTCT) of HIV infection [1] and reducing HIV-associated maternal morbidity and mortality [2]. Although ART initiation in pregnancy is important in promoting maternal and child health in the context of HIV/AIDS, there is growing evidence that only a fraction of eligible pregnant women receive ART before delivery [3], [4]. In most settings across sub-Saharan Africa, the design of health systems is a fundamental hurdle to ART in pregnancy [5]. HIV-infected eligible women are identified through PMTCT programmes within antenatal care (ANC), but ART initiation and follow-up typically takes place at separate HIV care and treatment services. Adult HIV care and treatment services are not well-oriented to the needs of HIV-infected pregnant women [6], leading to calls to integrate antenatal and HIV care and treatment services by providing ART within ANC services [7], [8].

Few studies have examined the impact of integrating ART delivery into ANC services, and the results of these are mixed. Findings from a systematic review of integrating ART into maternal and child health services suggest that integration results in higher ART enrolment and coverage, however, these findings were limited to four studies which fitted strict selection criteria and did not account for the variation in models of integrated care [9]. One study in Zambia demonstrated that providing ART within ANC increased the uptake of ART but did not have any effect on the time to initiation or retention in care [10]. Results from a study in Malawi also showed that even with a series of interventions to integrate services over three years, attrition and delays in referring pregnant women for ART were experienced [11]. Another South African study found that weekly provision of ART providers in ANC reduced the median time to ART initiation [12]. However, other studies have found no difference in the proportion of women initiating ART between integrated versus separate antenatal and ART services [13]. Together, this evidence demonstrates that integration of ART into ANC services may be feasible but does not necessarily improve maternal and child health outcomes. Furthermore, there is a general lack of consensus in health systems research regarding the defining criteria of integration [14], and models on the latter frequently vary in terms of their success in different contexts [9]. In turn, there is a clear need for operational research to understand the impacts integrating ANC and ART services [15].

Previously, we examined models of care for ART initiation during pregnancy in Cape Town, South Africa. In a cohort of women from 2005, we found no differences in ART initiation before delivery when comparing integrated versus separated services [3]. However, that study took place at a time when public sector ART services were relatively new in this setting, and it is possible that differences between integrated and separated services would only emerge over time as various models of care become more routine. To test this hypothesis, we investigated antenatal ART initiation among eligible pregnant women attending three different antenatal care services across Cape Town during 2008.

## Methods

We identified retrospectively a cohort of pregnant women who presented during the 2008 calendar year at three public sector primary care antenatal services. PMTCT services have been available in this setting from 2001 [16] and in 2004, PMTCT protocols were redrafted to include referral of pregnant women who were identified as ART-eligible to ART sites [17]. National guidelines at the time of this study recommended ART initiation in women with CD4 counts ≤200 cells/µL [18]. Over the course of 2008, the sites began to roll out ART to women with CD4 counts ≤250 cells/µL [19]. Women who were eligible for ART but who did not start antepartum may have received AZT during the antenatal period and later initiated ART postpartum.

### Services

The three participating services each implemented a different model for delivering ART to eligible pregnant women. The first (Site 1) consisted of an ‘integrated’ model in which women were able to initiate ART within the antenatal clinic on one specific day of the week when obstetricians with an HIV specialisation were on site; this model of care was set up and overseen by an international NGO supporting services in the district. The second (Site 2) we denoted a ‘proximal’ approach in which women were referred by letter to a separate ART service located within 100 metres of the maternity unit on the same premises. The third service model, the ‘distal’ approach (Site 3), delivered ART at a separate primary health care facility approximately three kilometres from the antenatal service, also using a referral letter. Each site delivered the same clinical services according to standard provincial ART protocols, which included same day CD4 cell count testing, which were processed at an external laboratory, with results being available at the antenatal service within a week. Although there was no active tracking system for the referral letter, psychosocial support was provided to eligible women by trained lay counsellors.

### Procedures

The cohort of ART-eligible pregnant women seeking care at each site during 2008 was assembled from clinical and laboratory records, linked by patients’ folder number, name and date of birth. HIV counselling and testing, HIV status and CD4 cell counts and obstetric data, came from routine service registers. In instances of missing data, electronic medical records systems were accessed for laboratory and obstetric information. To ascertain ART initiation and coverage among eligible women, the electronic and paper records of all 31 ART clinics (15 of which had electronic data at the time) in Cape Town were examined.

### Ethics Statement

Study approval was obtained from the University of Cape Town Human Research Ethics Committee (approval reference: 054/2007) and local government authorities, who approved the use of routinely collected services data and waived the need for written informed consent of those included in the cohort. The use of personal identifiers for linkage purposes was approved and deemed to be of minimal risk to patients attending the services. All data were kept confidential and stripped of unique identifiers after data collection was complete and the linkages had been made.

### Measures

The primary outcome was ART initiation before delivery. ART eligibility was based on a documented CD4 cell count during pregnancy of ≤200 cells/µL. Antenatal ART initiation was confirmed by an initiation date which fell between first antenatal presentation and delivery date or in the event of no available initiation date, affirmative evidence from the antenatal folder or labour ward register indicating ART coverage. In cases of a missing delivery date, antenatal ART initiation was considered affirmative if the ART initiation date was within 90 days of antenatal presentation, and postnatal initiation >90 days after antenatal presentation. This was based on a calculation of the average time between presentation and delivery in the cohort of HIV-infected women. ART coverage was defined as the proportion of all women on ART by the time of delivery, including women who initiated ART during pregnancy plus women already on ART at the time of presentation to antenatal care.

### Analysis

Data were analysed using Stata version 12 (STATA Corporation, College Station, USA). Proportions of women completing each step of the PMTCT cascade were estimated according to service delivery model. Bivariable associations were tested using Pearson’s Chi-squared test and Kruskal-Wallis test. Kaplan-Meier methods were used to calculate the proportion of eligible women initiating ART before delivery. Poisson regression with robust standard errors was used to examine whether the association between ART initiation and service delivery model (integrated/Site 1 vs proximal/Site 2 vs distal/Site 3) persisted after adjustment for potential confounding variables. An iterative modelling process was used to select and identify confounding variables in the model building process. Model results are expressed as rate ratios (RR) for antenatal ART initiation with 95% confidence intervals (CI).

## Results

**Figure 1:**
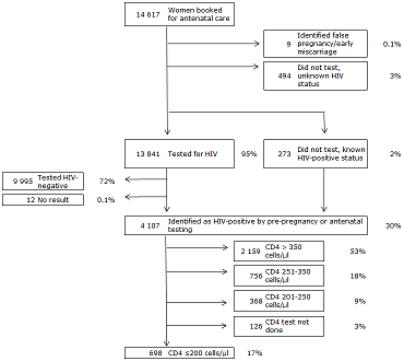
PMTCT Cascade depicting pregnant women accessing the three antenatal service models: HIV testing and ART eligibility.

A total of 14,617 women presented for antenatal care across the three antenatal services during 2008: 4879 at Site 1; 4990 at Site 2 and 4 748 at Site 3. Nine women were excluded from the analysis due to early pregnancy loss or false-positive pregnancy testing (Figure 1).

There were several significant differences in the characteristics of women seeking care at the three sites (Table 1). The median age at presentation was 25 years (Interquartile Range [IQR]: 22–30 years), with slightly older women attending Site 1. HIV counselling and testing uptake was high but varied between services, with Site 1 achieving higher uptake (99%) and Site 3 testing the lowest proportion of women (89%; p<0.001). At Site 1, 32% of women tested HIV-positive compared to 18% at Site 3 (p<0.001). Overall, 3% of women did not have a CD4 cell count result recorded either in paper or electronic sources, and this proportion did not vary across sites. The median CD4 cell count among all HIV-infected women was 373 cells/µL (IQR: 240–542), and 17% of all women had CD4≤200 cells/µL; this proportion varied between sites with the largest proportion of eligible women at Site 2 (p = 0.037, Table 2). Across all sites, a further 27% of HIV-infected women had a CD4 cell count of 200–350 cells/µL (Table 2).

**Table 1:** Descriptive characteristics of the cohort of women presenting at the three service models in 2008.

	Site 1 (integrated)		Site 2 (proximal)		Site 3 (distal)		P-value*	Total	
	n	%/IQR	n	%/IQR	n	%/IQR		n	%/IQR
**All women presenting for antenatal care**	**4,879**	**33.4**	**4,990**	**34.1**	**4,748**	**32.5**		**14,617**	
Total excluding women who were not pregnant or identified as experiencing miscarriage	**4,877**	**33.4**	**4,987**	**34.1**	**4,744**	**32.5**		**14,608**	
Median age in years at 1^st^ presentation (IQR)	27	(23–31)	25	(21–30)	25	(21–30)	<0.001	25	(22–30)
Tested (% of all women)	4,864	99.7	4,725	94.8	4,252	89.6	<0.001	13,841	94.7%
HIV-prevalence (all)	1,609	32.1	1,458	29.2	1,040	21.9	<0.001	4,107	29.7%
HIV prevalence (among those who tested)	1,599	32.9	1,458	30.9	777	18.3	<0.001	3,834	28.1%
Median CD4 cell count (IQR)	382	(252–561)	372	(231–538)	362	(242–532)	0.05	373	(241–542)
Missing CD4 cell count	53	3.3	30	2.1	43	4.1	0.01	126	3.0%
CD4≤200 cells/µl (% of tested HIV-positive)	252	15.6	277	19.0	169	16.2	reference	698	17.0%
CD4 201–250 cells/µl (% of tested HIV-positive)	134	8.3	141	9.7	93	8.9	0.769	368	9.0%
CD4 251–350 cells/µl (% of tested HIV-positive)	307	19.1	238	16.3	211	20.3	0.004	756	18.4%
CD4>350 cells/µl (% of tested HIV-positive)	865	53.7	773	53.0	525	50.3	0.040	2,159	52.6%

* Bivariable associations derived using Kruskall-Wallis and Pearson chi-squared tests.

**Table 2:** Descriptive characteristics of women with CD4 counts ≤200 cells/µL.

	Model 1 (integrated)		Model 2 (proximal)		Model 3 (distal)		P-value*	Total	
**Women with CD4 cell counts ≤200 cells/µl**	**252**	**36.1%**	**277**	**39.7%**	**169**	**24.2%**	**0.037**	**698**	
Median age at 1st presentation (SD)	29	(24–32)	28	(24–32)	28	(24–32)	0.3708	28	(24–32)
Median gestational age in weeks at 1st presentation (557/698 women with available data, IQR)	26	(21–32)	27	(21–32)	23	(19–28)	<0.001	26	(21–31)
Nulliparous (522/698 women with available data)	63	12.1%	73	14.0%	40	7.7%	0.646	176	
Median CD4 cell count (cells/µl, IQR)	144	(106–172)	139	(103–167)	145	(99–175)	0.2632	142	(103–171)
Initiated ART before antenatal presentation	37	14.7%	30	10.8%	14	8.3%	0.048	81	11.6%
**Eligible women who had not initiated ART before antenatal presentation**	**215**	**85.3%**	**247**	**89.2%**	**155**	**91.7%**		**617**	**88.4%**
Initiated Antenatal ART (% of remaining eligible women who had not initiated beforeantenatal presentation)	120	55.8%	94	38.1%	70	45.2%	0.003	284	46.0%
Did not initiate antenatal ART (% of remaining women who had not initiated before antenatal presentation)	77	35.8%	115	46.6%	64	41.3%		256	41.5%
Unknown (Eligible by CD4 test result, patient folder missing)	18	8.4%	38	15.4%	21	13.5%		77	12.5%
No documentation of antenatal ART (% of remaining eligible women who were notdocumented as being on ART by delivery)	95	44.2%	153	61.9%	85	54.8%		333	54.0%
Received single or dual prophylaxis (% of women who did not receive ART antenatally orwhose intervention status was unknown)	40	18.6%	58	23.5%	51	32.9%	0.015	149	24.1%
Initiated ART postpartum	46	21.4%	50	20.2%	29	18.7%	0.228	125	20.3%
**ART coverage at delivery (% of all women who were on ART at delivery)**	**157**	**62.3%**	**124**	**44.8%**	**84**	**49.7%**	**0.001**	**365**	**52.3%**
Median gestational age in weeks at 1st presentation (500/617 women with available data, IQR)	26	(21–31)	27	(21–32)	23	(19–29)	<0.001	26	(21–31)
Median gestational age at ART initiation (of 164/284 women with available data, IQR)	31	(28–34)	31	(28–34)	30	(27–34)	0.946	31	(28–34)

* Bivariable associations derived using Kruskall-Wallis and Pearson Chi 2 tests

### ART Initiation in Eligible Women

Of the 658 women with a CD4 cell count ≤200 cells/µL at the three sites, 11% (n = 81) were already on ART at their first antenatal visit. This proportion was significantly higher at Site 1 (14%) compared to the other sites (p = 0.04). When excluding women on ART at presentation, the overall percentage of eligible women who were initiated on ART during pregnancy was 46%. There was a significant difference in the proportions of women who initiated ART between the sites, with 55%, 38% and 45% of eligible women initiating ART during pregnancy at sites 1 (integrated), 2 (proximal), and 3 (distal), respectively (global p = 0.003). The remaining 333 women (54%) had no documentation of antenatal ART initiation (comprising 42% of women who did not initiate in pregnancy according to the record review, and 13% of women for whom records were missing) Among the women initiating ART during pregnancy, 5% of women presenting at Site 1 initiated at an ART service other than the designated referral site, compared to 14% of women from Site 2 and 22% of women from Site 3 (Table 2).

### Gestational Age at First Presentation

The estimated median gestational age at first antenatal presentation among all ART-eligible women was 26 weeks (IQR: 21–31 weeks) and varied significantly between the sites, with women from Site 3 presenting at 23 weeks and women from Sites 1 and 2 presenting at 26 and 27 weeks, respectively (p<0.001). Across all sites, women who initiated ART presented at a significantly earlier gestational age (23 weeks) compared to women who did not start ART during pregnancy (29 weeks; p<0.001). However, the median gestational age at the time of antenatal ART initiation (31 weeks; IQR, 28–34 weeks) did not vary by site (p = 0.946,Table 2).

In women who presented at or after 32 weeks’ gestation, there was little difference in the proportion of women initiating ART between sites (5%–6% across sites; p = 0.435). Instead, the overall differences between sites were observed in women who presented before 32 weeks. At Site 1, 36% of all eligible women who presented before 32 weeks’ gestation were initiated, while Site 2 initiated 27% and Site 3 initiated 20% of these women, respectively.

### Delays to Treatment Initiation

**Figure 2:**
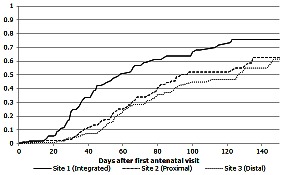
Kaplan-Meier failure estimates of time to treatment in women who initiated ART in pregnancy.

Figure 2 shows the proportions of eligible pregnant women initiating ART over time prior to delivery. Overall, half of women started ART within 49 days of their first antenatal care visit (IQR: 31–67 days). The median time to treatment initiation was significantly lower at Site 1 (36 days) compared to Sites 2 and 3 (54 and 59 days, respectively) (p<0.001). There was no significant difference in the median time to treatment initiation by CD4 cell count (log-rank p = 0.503 comparing CD4 cell count ≤100 cells/µL versus 101–200 cells/µL). In addition, a further 20% (125/617) of eligible women who did not receive ART in pregnancy went on to initiate up to 3 years postpartum. The median time of ART initiation in this group was 34 weeks after delivery; the time to postpartum initiation was not significantly associated with the antenatal site (p = 0.149).

### Characteristics of Women who did and did not Initiate ART Antenatally

In a regression model predicting antenatal ART initiation (Table 3), women who attended Site 1 (integrated model) were significantly more likely to initiate ART in pregnancy (RR: 1.33; 95% CI: 1.09–1.64) compared to women attending Site 3 (distal model) after adjusting for covariation in gestational age at booking and maternal age. There was no difference in the probability of antenatal ART initiation comparing Sites 2 and 3, however (p = 0.704). In addition, increasing gestational age at first antenatal presentation was associated with decreased probability of antenatal ART initiation. Compared to women presenting at or before 20 weeks’ gestational age, women presenting at 25–28, 29–32, 33–36 and after 36 weeks’ gestational age were 28%, 38%, 78% and 82% less likely to start ART during the antenatal period. Increasing maternal age was also associated with increasing probability of antenatal ART initiation (RR for a 1-year increase in age, 1.02; 95% CI: 1.00–1.03).

**Table 3:** Poisson regression predicting the probability of antenatal ART initiation among eligible pregnant women.

Variable	Rate Ratio	p-value	95% confidence interval
**Service delivery model**			
Site 3	Reference		
Site 2	1.05	0.704	(0.83–1.31)
Site 1	1.34	0.005	(1.09–1.64)
**Gestational age at presentation**			
≤20 weeks	Reference		
21–24 weeks	0.94	0.543	(0.78–1.14)
25–28 weeks	0.72	0.002	(0.58–0.88)
29–32 weeks	0.62	<0.001	(0.48–0.79)
33–36 weeks	0.22	<0.001	(0.13–0.38)
36–40 weeks	0.18	0.002	(0.06–0.54)
**Maternal age**	1.02	0.04	(1.00–1.03)

## Discussion

This study examined antenatal ART initiation in eligible pregnant women in Cape Town during 2008. Although overall levels of ART initiation were relatively low, the findings suggest that an integrated model of antenatal ART initiation may be associated with higher ART uptake compared to models that separate ANC and ART services.

The proportion of women starting ART in pregnancy in these data from 2008 (46%) is slightly lower than the corresponding proportion from the same facilities in 2005 (51%). These persistently low levels of antenatal ART initiation point to the ongoing challenges in starting ART during pregnancy. Health services for ART initiation in adults expanded in Cape Town between 2005 and 2008 (doubling from 33 clinics in 2005 to 66 in 2009). However, these general ART services are often not oriented to the needs of pregnant women. First, pregnant women tend to be clinically stable compared to other eligible adults [20], [21] and may not receive adequate attention in the general pool of more morbid patients initiating treatment. Second, there are unique psychosocial barriers facing pregnant women starting ART which receive little attention in routine ART counselling models [22].

Here, the integrated model for starting ART in pregnancy saw a higher percentage of women initiating ART before delivery. In this model, the vast majority of women (95%) who started ART antenatally did so within the ANC, compared to lower proportions in the other models with referral ART services. Previous studies have reported similarly low rates of ART initiation among women referred to centralised ART services [4], [23]. It may be possible that with increased distance between antenatal and ART services, factors such as convenience or desire for privacy may adversely affect referral and uptake of ART. For example, qualitative research from Malawi has suggested that pregnant women have a preference for integrated ART services over access to ART in general primary level services where they would be required to mix with HIV-infected men and non-pregnant women with more advanced HIV disease [24].

These findings also demonstrate that regardless of the model of care involved, late antenatal presentation is a persistent barrier to antenatal ART initiation. In this setting, women presented for care in pregnancy into the second and third trimesters, decreasing the time available for antenatal ART initiation. The phenomenon of late antenatal presentation is a well-known concern in maternal and child health [25]–[27], and our findings are consistent with other studies [28], [29]. The frequency of late antenatal presentation in this and other settings means that expediting ART initiation in eligible women is critical, as are complementary efforts to encourage women to attend antenatal care earlier [30]. The median time to treatment in women who initiated postpartum was 34 weeks, which may suggest identification and linkage to care through baby immunization services. More research is required to understand the reasons for delayed initiation in these women who were not lost to the service. Under the integrated model of care, women started ART more quickly (median delay from antenatal presentation to ART initiation, 36 days) than in other models (median delay, 54 and 59 days at Sites 2 and 3, respectively). Several studies have suggested that each additional week of ART provided before delivery results in a significant reduction in the risk of vertical transmission [31] pointing to additional potential benefits to an integrated model of care for PMTCT.

Our findings are particularly important given the interest in universal ART initiation for all HIV-infected pregnant women, regardless of CD4 cell count [32]. Implementation of the World Health Organization’s “Option B+” strategy would dramatically increase the numbers of women eligible to start lifelong ART in pregnancy in South Africa and other high-prevalence settings, and would also require new service delivery approaches that can assist in starting ART as quickly as possible during pregnancy. Integration of ANC and ART services presents one valuable strategy to achieve these aims. However, it is important to note that this research focuses on ART initiation, and there are separate concerns regarding treatment adherence and retention in care among women starting ART during pregnancy [33]. These issues are likely to persist across models of care, and will require specific attention in the design and operation of integrated or separated ART-ANC services.

The interpretation of these data comes with several limitations. There may be important differences between the three service delivery models other than their approach to antenatal ART initiation and thus it is difficult to infer that the increased antenatal ART initiation at Site 1 is attributable solely to the integration of services. For example, we did not assess whether the time for treatment workup differed between the models. Each ART eligible woman would have required both clinical and psychosocial assessment prior to initiation and it is possible that approaches varied between the models and within the sites depending on service provider. This may have negatively impacted on women who presented in late pregnancy in particular, due to there being little time for work up. Despite this, guidelines did not preclude women in advanced pregnancy (>36 weeks gestation) from initiating ART. Related to this, it is important to note that definitions of service integration related to HIV/AIDS and reproductive health vary widely [34]. The integration studied here may not be appropriate in all settings and alternative approaches to integration may be more relevant in other health systems contexts; further research into integrated models of ANC and ART is warranted.

This research was conducted in an urban setting with a high antenatal HIV prevalence and high-volume ANC and ART services, and the findings should be generalized with caution. This analysis was conducted under previous WHO guidelines (with a CD4 threshold for ART initiation of 200 cells/uL) and the numbers of women requiring ART have almost doubled since the implementation of the 2010 WHO guidelines; it is unclear whether such increases would alter the differences shown here between integrated and separated services. In addition, these data come from a retrospective review of clinical records, and hence the accuracy and completeness data may be suboptimal, though there were no differences in the levels of missing data between service delivery models.

In summary, with the efficacy of drug regimens for PMTCT well-established, PMTCT programme impact is dependent on the implementation of appropriate and effective service delivery models [35]. This study suggests that integration of ART into routine antenatal care services can lead to significant improvements in ART initiation during pregnancy, increasing both the proportion of eligible women who start ART and the duration of ART received before delivery. While further research is required, integration of ART into antenatal care represents a valuable approach for promoting maternal and child health in the context of HIV/AIDS.
